# Association between maternal antibiotic exposure during pregnancy and childhood obesity in the Japan Environment and Children's Study

**DOI:** 10.1111/ijpo.12956

**Published:** 2022-06-24

**Authors:** Kenichi Sakurai, Midori Yamamoto, Akifumi Eguchi, Rieko Takatani, Masahiro Watanabe, Chisato Mori, the Japan Environment and Children's Study Group

**Affiliations:** ^1^ Department of Nutrition and Metabolic Medicine, Center for Preventive Medical Sciences Chiba University Chiba Japan; ^2^ Department of Sustainable Health Science, Center for Preventive Medical Sciences Chiba University Chiba Japan; ^3^ Department of Bioenvironmental Medicine, Graduate School of Medicine Chiba University Chiba Japan

**Keywords:** antibiotics, childhood obesity, pregnancy

## Abstract

**Background:**

The association between maternal antibiotic exposure during pregnancy and childhood obesity is still unclear.

**Objectives:**

The study aimed to evaluate the association between prenatal exposure to antibiotics and obesity at age 3 years using data from a large Japanese birth cohort.

**Methods:**

The Japan Environment and Children's Study is a nationwide birth cohort study. In this study, singleton vaginal full‐term births were included. Obesity was defined as body mass index ≥95th percentile according to child growth standards. Prenatal antibiotic exposure was defined as antimicrobial agent use during pregnancy and was collected from maternal interviews and medical record transcripts. Logistic regression analysis was performed to evaluate the association of prenatal antibiotic exposure with child obesity at 3 years.

**Results:**

In the crude and adjusted models with all children, maternal antibiotic exposure during pregnancy showed a marginal relationship with child obesity at 3 years. In the analyses according to exposure period and sex, exposure to antibiotics during the second/third trimester was significantly associated with obesity at the age of 3 years in female infants, but not in male infants, although the exposure during the first trimester was not in both sexes.

**Conclusion:**

Maternal antibiotic exposure during mid/late pregnancy may result in child obesity.

## 1 INTRODUCTION

The prevalence of obesity is increasing at an alarming rate worldwide.^1^, ^2^ Obesity causes metabolic and cardiovascular diseases, such as metabolic syndrome, type 2 diabetes mellitus, and ischaemic heart disease, and is associated with all‐cause mortality.^3^ Prevention of obesity is required to reduce the risks of these subsequent events.

To prevent obesity in adulthood, it is important to also consider childhood obesity. According to the obesity trajectory, the onset of obesity at a younger age has been reported to be associated with a longer obesity duration. Subsequently, a longer duration of obesity results in increased cardiometabolic risks.^4^ The World Health Organization has also raised the alarm and stated that children who are overweight or obese are more likely to still be affected by obesity in adulthood and develop non‐communicable diseases, such as diabetes and cardiovascular diseases, at a younger age.^5^ Therefore, the prevention of childhood obesity is one of the most important strategies against an epidemic of obesity and cardiovascular risks.

As per the concept of the developmental origins of health and disease,^6^ the fetal environment is thought to affect diseases later in life, such as ischaemic heart disease, metabolic syndrome, type 2 diabetes mellitus, and other non‐communicable diseases.^7^ Thus, it is important to understand the effects of the fetal environment on childhood obesity and subsequent adult obesity.

Recently, the gut microbiota has been shown to play an important role in human health, including obesity,^8^ and people with obesity were reported to have a different gut microbiota composition compared with people without obesity.^9^, ^10^, ^11^ Moreover, mice that underwent faecal transplantation from people with obesity developed an obesity phenotype. Gut microbiota may be disturbed by many factors, including antibiotics.^12^ Antibiotic use causes dysbiosis in both experimental animals and humans.^13^ Indeed, antibiotic prescription was associated with obesity prevalence in the United States,^14^ and mouse models of dysbiosis developed an obesity phenotype.^15^

Furthermore, antibiotic exposure in infancy was reported to affect childhood obesity.^16^, ^17^ Antibiotic exposure before 6 months of age, or repeatedly during infancy, was associated with increased body mass index (BMI) at age 24 months.^18^ Poulsen et al. reported that prenatal antibiotic use was not associated with the child's BMI‐for‐age z‐score, but early life exposure to antibiotics was.^19^ However, other studies have shown that maternal exposure to antibiotics during pregnancy was associated with obesity in their children.^20^, ^21^ Several studies have indicated the importance of the types and dosage of antibiotics used or the presence of coexisting infection for being overweight during childhood.^22^, ^23^, ^24^ A meta‐analysis reported that prenatal exposure to antibiotics was not significantly associated with childhood overweight or obesity, whereas an increased risk of overweight or obesity was seen in a subgroup analysis of maternal antibiotic use in the second trimester of pregnancy.^25^ The association between prenatal exposure to antibiotics and childhood obesity is therefore still under discussion. This study aimed to evaluate the association between prenatal exposure to antibiotics and obesity at the age of 3 years using data from a large Japanese birth cohort.

## 2 METHODS

### 2.1 Study population

The Japan Environment and Children's Study (JECS) is a nationwide government‐funded birth cohort study, and its study design and baseline profile have been previously described elsewhere.^26^, ^27^ The JECS cohort was recruited from pregnant mothers from study areas throughout Japan from January 2011 to March 2014; in total, 103 060 pregnancy events were enrolled. This study was based on the dataset of jecs‐ta‐20 190 930, which was released in October 2019. For this study, the data collected comprised antibiotic use in pregnancy as the exposure and infant BMI at the age of 3 years as the outcome.

In this study, singleton vaginal full‐term births were included. Exclusion criteria were children born by caesarean section and without information about antibiotic use in medical record transcripts. Participants without anthropometric data at the age of 3 years were also excluded from the analyses.

### 2.2 Ethics

The JECS protocol was reviewed and approved by the Institutional Review Board on Epidemiological Studies of the Ministry of the Environment and by the Ethics Committees of all participating institutions. The JECS was conducted in accordance with the Helsinki Declaration and other nationally valid regulations and guidelines. Written informed consent was obtained from all participants.

### 2.3 Anthropometry at 3 years

Body height and weight at 3 years measured at a local health care center, a medical institute, a nursery facility, home, or other location were included in the questionnaires at 3 years after birth. Body weight was measured in underclothes and without shoes, and height was measured without shoes. The BMI (in kg/m^2^) was calculated as follows: (body weight: kg)/(height: m)^2^. Obesity was defined as a BMI‐for‐age ≥ 95th percentile according to the Japanese child growth curves.^28^

### 2.4 Antibiotic exposure

Information on prenatal antibiotic exposure, which was defined as an antimicrobial agent intake during pregnancy, was collected by (1) interview questionnaire answered by mothers upon enrollment (InT1) and during mid‐late pregnancy (InT2) and (2) review of medical records that were transcribed after delivery by physicians, midwives/nurses, and Research Co‐ordinators. In the former, the answers of the mothers on medication were recorded by Research Co‐ordinators according to a medication list. This study used only the data on antibiotic exposure after confirmation of pregnancy. For the review of medical record transcripts, exposure to antibiotics was designated when the word ‘antibiotics’ or any antibiotic name was described as a medication during pregnancy. Antibiotic exposure was defined as systemic antibiotic use (orally or by injection). The use of local antibiotics in the form of ointments, gels, or drops was not included in this analysis. The logistic regression analysis combined data from both the interview questionnaire and the medical record transcripts to define antibiotic exposure during pregnancy (see methods below).

The interview questionnaire used in this study asked mothers: ‘I took medicine/drug/supplements in the past year’. Then, participants who answered ‘Yes’ were asked about the period during which they took the medication (‘During the first trimester’ and/or ‘Between the beginning of the second trimester and now’) and the type of medication (‘Antibacterial drugs’). The time of antibiotic exposure was divided into the first (T1) and second or third (T2/3) trimester according to the interview questionnaire. The effects of the time of antibiotic exposure were analysed using this classification.

### 2.5 Other variables

Based on previous JECS publications on the children's body weight, the following covariates were selected and included in the multivariate analysis: maternal age at delivery, annual household income, marital status, parity, pre‐pregnancy BMI, any complication during pregnancy or delivery, and the sex of the infant. Full‐term birth was defined as gestational age at birth of ≥37 and <42 weeks.

### 2.6 Statistical analysis

Multiple logistic regression was performed to estimate the odds ratio for obesity at 3 years and antibiotic exposure during pregnancy, with adjustments for all other covariates. The forced entry method was used to include all covariates. Numerical variables were included in the analytical models as continuous variables. *p*‐values <0.05 were considered statistically significant. All analyses were conducted using R version 3.6.0 (2019‐04‐26) (R Foundation for Statistical Computing, Vienna, Austria; https://www.R-project.org/) with the ‘psych’ package.^29^

## 3 RESULTS

Among the 100 304 registered live births, 56 416 participating children without exclusion criteria were analysed (Table S1). In total, 15 980 exposed cases and 40 436 controls were identified and included in the analyses.

The characteristics of the study participants are shown in Table 1. Mean maternal age at delivery was about 31 years in both groups. Mean maternal pre‐pregnancy BMI was 20.95 and 21.04 kg/m^2^ in the unexposed and exposed groups, respectively. The mean infant BMI at 3 years was 15.98 and 16.03 kg/m^2^ in the unexposed and exposed groups, respectively.

**TABLE 1:** Characteristics of study participants

Antibiotics use during pregnancy	(−)	(+)	Effect size
*N*	40 436	15 980	
Maternal height (cm)	158.40 (5.24)	158.37 (5.24)	0.0072^a^
Maternal weight (kg)	52.60 (8.10)	52.82 (8.46)	−0.0267^a^
Pre‐pregnancy BMI (kg/m^2^)	20.95 (2.96)	21.04 (3.07)	−0.0310^a^
Maternal age at delivery (years)	31.27 (4.78)	31.08 (4.91)	0.0397^a^
Child sex (male/female)	20 544/19892 (50.8/49.2)	8159/7821 (51.1/48.9)	0.000^b^
Height at 3 years (cm)	91.74 (3.70)	91.69 (3.71)	0.0116^a^
Weight at 3 years (kg)	13.47 (1.47)	13.49 (1.45)	−0.0140^a^
BMI at 3 years (kg/m^2^)	15.98 (1.25)	16.03 (1.27)	−0.0345^a^

*Note*: Data are presented as mean (standard deviation) or number (%).

Abbreviation: BMI, body mass index.

^a^ Cohen's *d* value.

^b^ phi coefficient.

The number of children with obesity was 5304, which amounted to 9.40% of the included participants. The percentage of cases with obesity was 9.25% and 9.78% in the unexposed and exposed groups, respectively. Table 2 summarizes the results of the univariate and multivariate analyses for the association between childhood obesity and maternal antibiotic exposure during pregnancy. In the univariate analyses, antibiotic exposure was marginally associated with childhood obesity. However, the association was not shown after adjustment with covariates.

**TABLE 2:** Results of logistic regression analysis on the effects of antibiotic exposure during pregnancy on obesity at 3 years of age

	Sample size	Odds ratio	95% CI	*p*‐value
Crude	56 415	1.06	1.00–1.13	0.052
Adjusted^a^	55 911	1.04	0.98–1.11	0.202

Abbreviation: CI, confidence interval.

^a^ Adjusted according to maternal age at delivery, pre‐pregnancy body mass index, household income, gestational age at birth, birth weight, diabetes mellitus, and gestational diabetes mellitus.

In our previous study, the effects of the gut microbiome on the head circumference at birth were different between males and females.^30^ Thus, we evaluated the relationship between maternal antibiotic exposure and the offspring's obesity according to infant sex.

In female infants, the BMI at 3 years of age was 15.91 and 15.96 kg/m^2^ in the unexposed and exposed groups, respectively. The percentage of infants with obesity was 8.77% and 9.56% in the unexposed and exposed groups, respectively. In male infants, the mean BMI at 3 years of age was 16.06 and 16.09 kg/m^2^ in the unexposed and exposed groups, respectively. The percentage of cases with obesity was 9.72% and 9.99% in the unexposed and exposed groups, respectively.

Table 3 shows the results of the logistic regression analyses, which were performed for each sex separately. The results showed that maternal antibiotic exposure during pregnancy was significantly associated with childhood obesity in the female crude model. After adjustment, however, this association was no longer significant. In contrast, maternal antibiotic exposure during pregnancy was not significantly associated with childhood obesity in males in both the crude and adjusted models.

**TABLE 3:** Results of logistic regression analysis on the effects of antibiotic exposure during pregnancy on obesity at 3 years of age according to sex

	Male				Female			
	Sample size	Odds ratio	95% CI	*p*‐value	Sample size	Odds ratio	95% CI	*p*‐value
Crude	28 703	1.03	0.95–1.12	0.490	27 713	1.10	1.01–1.20	0.037
Adjusted^a^	28 447	1.03	0.94–1.13	0.466	27 424	1.05	0.96–1.16	0.293

Abbreviation: CI, confidence interval.

^a^ Adjusted according to maternal age at delivery, pre‐pregnancy body mass index, household income, gestational age at birth, birth weight, diabetes mellitus, and gestational diabetes mellitus.

Next, the effects of antibiotic exposure on obesity at the age of 3 years were examined according to the exposure period, divided into the first or the second/third trimesters, since we did not ask the time of exposure separately for the second and third trimester. Antibiotic exposure during the first trimester was not significantly associated with obesity at the age of 3 years in males and females. However, antibiotic exposure during the second/third trimesters was significantly associated with obesity at the age of 3 years in females, but not in males. This association remained significant in females after adjustment (Table 4).

**TABLE 4:** Results of logistic regression analysis on the effects of antibiotic exposure on obesity at 3 years of age according to the exposure period

		Male				Female			
		Sample size	Odds ratio	95% CI	*p*‐value	Sample size	Odds ratio	95% CI	*p*‐value
Before 12 weeks^a^	Crude	18 711	1.07	0.86–1.33	0.536	18 220	1.12	0.89–1.41	0.327
	Adjusted^b^	18 531	1.10	0.87–1.37	0.432	18 060	0.99	0.77–1.27	0.907
After 12 weeks^a^	Crude	21 716	1.11	0.97–1.27	0.15	21 063	1.27	1.11–1.46	0.001
	Adjusted^b^	21 517	1.12	0.98–1.30	0.10	20 887	1.23	1.06–1.42	0.007

Abbreviation: CI, confidence interval.

^a^ Gestational age.

^b^ Adjusted according to maternal age at delivery, pre‐pregnancy body mass index, household income, gestational age at birth, birth weight, diabetes mellitus, and gestational diabetes mellitus.

## 4 DISCUSSION

In this study, the association between maternal antibiotic exposure during pregnancy and the offspring's obesity at the age of 3 years was evaluated. The results indicated that maternal exposure to antibiotics during the second or third trimester was associated with obesity at 3 years in female infants; however, the association was not observed in male infants.

Maternal exposure to antibiotics during pregnancy has been reported to be associated with several childhood health problems in physical and mental health, such as asthma^31^ or mood and anxiety disorders.^32^ The prescription of antibiotics has been correlated with an increased prevalence of obesity in the United States.^14^ Childhood obesity has also been reportedly associated with antibiotic exposure during the fetal period or early childhood.^18^ Our results showed the association between fetal exposure to antibiotics and childhood obesity. These results suggest that early exposure to antibiotics, such as in the fetal period or early childhood, could play an important role in establishing childhood obesity.

Antibiotics cause dysbiosis, defined as a disturbed or imbalanced gut microbiota.^33^, ^34^ Recent studies have indicated that dysbiosis is one of the causes of obesity.^34^ It has been reported that people with obesity have different gut microbiota from people without obesity, and faecal transplantation from people with obesity could cause obese phenotypes in mice.^11^ Gut microbiota before and after antibiotic use showed different constitutions from each other.^35^ Maternal exposure to antibiotics during pregnancy changes the mother's gut microbiota, which in turn may affect the establishment of their offspring's gut microbiota.^36^ It is therefore possible that maternal dysbiosis causes child dysbiosis. The study hypothesis was that maternal antibiotic exposure during pregnancy results in the offspring's obesity through their affected gut microbiota. However, our results indicated little association between maternal antibiotic exposure and obesity at the age of 3 years. There is another possible mechanism linking maternal antibiotic exposure and the offspring's obesity. Kimura et al. reported that maternal gut microbiota affected the offspring's metabolic phenotype through short‐chain fatty acids derived from gut microbiota^37^; hence, it is necessary to consider this type of effect. Gut microbiota can also be affected by diet, lifestyle, and microbe exposure after birth.^38^, ^39^ For example, breastfeeding has been shown to optimize gut function in the early postnatal period, whereas when other liquids are introduced, the gut function is reduced.^40^, ^41^ There is also a link between early diet and later gut microbiota and body composition.^42^ Suboptimal infant feeding may also cause obesity later in life. It is therefore important to assess the relationship between these postnatal factors and obesity, as well as the gut microbiota.

The type of antibiotic used, dosage, and duration of antibiotic treatment during pregnancy are important factors that may predict obesity in children. Wang et al. examined the effect of repeated prenatal antibiotic exposure on childhood obesity. Their results indicated that repeated use of antibiotics during pregnancy had an increased risk of childhood obesity.^23^ Jess et al. showed that prenatal use of narrow‐ and broad‐spectrum antibiotics had different effects on childhood obesity.^22^ Our study did not collect the type, dosage, or duration of antibiotic use during pregnancy, so we did not examine these factors.

The effects of maternal antibiotic use were different between the exposure periods. One meta‐analysis reported that the effects of maternal antibiotic exposure during pregnancy on childhood obesity were different between the first, second, and third trimesters. The authors showed that antibiotic exposure during the second trimester was associated with childhood obesity but not during the first or third trimester.^25^ Although in our study, the exposure period was not divided into the second and third trimesters, exposure during the latter period of pregnancy was associated with obesity at the age of 3 years. These results suggest that antibiotic exposure during late pregnancy could be a risk factor for childhood obesity. However, the reason for these results is not clear. It has been reported that exposure to other factors, such as maternal nutrition and smoking,^43^, ^44^ affects birth weight, which has been associated with obesity in infancy. In the present study, birth weight was not significantly different between offspring with and without maternal antibiotic exposure. Maternal antibiotic exposure during pregnancy is suggested to affect child obesity through factors other than birth weight. A study using a humanized mouse model showed that the gut microbiota recovers from alterations caused by antibiotic treatment after some time.^35^ The effect of maternal antibiotic exposure close to the time of delivery may remain at delivery and affect their offspring. However, this theory is unlikely since Wan et al.^25^ reported that only antibiotic use during the second trimester was significantly associated with childhood obesity and not in the third trimester. There may, therefore, be a sensitive time window to antibiotic exposure during the fetal period that results in childhood obesity.

Our results indicate that the effects of maternal antibiotic exposure during pregnancy are different between males and females. Previously, we reported that maternal gut microbiota during pregnancy was associated with the newborn's head circumference and that this association was different between males and females.^30^ We also reported that the association between the umbilical serum level of mercury and DNA methylation of umbilical cord tissue was different between both sexes.^45^ Male and female offspring show several differences in their blood and tissues.^46^ Recently, it was reported that the effects of gut microbiota on metabolism or gene expression were different between males and females in mouse models.^47^, ^48^, ^49^ It is therefore possible that maternal antibiotic exposure during pregnancy affects their offspring via the child's gut microbiota in a sex‐specific manner. Fetal exposure to various factors may cause different effects in males and females. In the present study, these precise mechanisms have not been clarified, and further study is needed in this regard.

### 4.1 Limitations

There are several limitations to this study. The timing of antibiotic exposure during pregnancy was obtained by medical record transcripts and self‐reported questionnaires, and the latter may cause recall bias. In this study, the type of antibiotic and duration of use were not identified; hence, these differences were not analysed. The reasons for antibiotic use during pregnancy may affect the offspring's obesity, but these data were not obtained. Antibiotics used to treat infectious diseases have been reported to affect childhood obesity.^24^ Although we should consider the coexisting infection with antibiotic use, we did not have any data about maternal infection. It is, therefore, a limitation to the translation of our results.

Furthermore, postnatal factors that can affect obesity were not evaluated. As one of the postnatal factors, infant nutrition by breastfeeding and formula feeding differentially affect childhood obesity.^50^ Another factor that also affects childhood obesity is antibiotic use after birth.^51^ Thus, the interaction between the postnatal factors and maternal antibiotic exposure during pregnancy should be considered in future studies.

## 5 CONCLUSION

Maternal antibiotic exposure during mid/late pregnancy marginally affects childhood obesity with sex dimorphism, although the findings of this study did not indicate the need for any change in the use of antibiotics when medically necessary. Further studies, including the assessment of gut microbiota, are needed to clarify this mechanism, which may provide a strategy to prevent childhood obesity.

## AUTHOR CONTRIBUTIONS

Kenichi Sakurai and Chisato Mori conceptualized and designed the study. Kenichi Sakurai, Midori Yamamoto, Masahiro Watanabe, and Akifumi Eguchi designed the analysis strategy; Kenichi Sakurai and Akifumi Eguchi analysed the data; Kenichi Sakurai, Midori Yamamoto, Rieko Takatani, and Akifumi Eguchi interpreted the data; and Kenichi Sakurai drafted the manuscript. All authors revised and approved the final draft.

## CONFLICT OF INTEREST

No conflict of interest was declared.

**Abbreviations**
BMI: body mass index
JECS: Japan Environment and Children's Study

## Supporting information


**Table S1.** Characteristics of cohort participants

## APPENDIX A

**Members of the JECS Group as of 2021**: Michihiro Kamijima (principal investigator, Nagoya City University, Nagoya, Japan), Shin Yamazaki (National Institute for Environmental Studies, Tsukuba, Japan), Yukihiro Ohya (National Center for Child Health and Development, Tokyo, Japan), Reiko Kishi (Hokkaido University, Sapporo, Japan), Nobuo Yaegashi (Tohoku University, Sendai, Japan), Koichi Hashimoto (Fukushima Medical University, Fukushima, Japan), Chisato Mori (Chiba University, Chiba, Japan), Shuichi Ito (Yokohama City University, Yokohama, Japan), Zentaro Yamagata (University of Yamanashi, Chuo, Japan), Hidekuni Inadera (University of Toyama, Toyama, Japan), Takeo Nakayama (Kyoto University, Kyoto, Japan), Hiroyasu Iso (Osaka University, Suita, Japan), Masayuki Shima (Hyogo College of Medicine, Nishinomiya, Japan), Youichi Kurozawa (Tottori University, Yonago, Japan), Narufumi Suganuma (Kochi University, Nankoku, Japan), Koichi Kusuhara (University of Occupational and Environmental Health, Kitakyushu, Japan), and Takahiko Katoh (Kumamoto University, Kumamoto, Japan).

## References

[1] Jaacks LM , Vandevijvere S , Pan A , et al. The obesity transition: stages of the global epidemic. Lancet Diabetes Endocrinol. 2019;7(3):231‐240. 30704950 10.1016/S2213-8587(19)30026-9 PMC7360432

[2] Obesity. World Health Organization. https://www.who.int/health-topics/obesity#tab=tab_1. Accessed January 26, 2021.

[3] Global BMIMC , Di Angelantonio E , Bhupathiraju SN , et al. Body‐mass index and all‐cause mortality: individual‐participant‐data meta‐analysis of 239 prospective studies in four continents. Lancet. 2016;388(10046):776‐786. 27423262 10.1016/S0140-6736(16)30175-1 PMC4995441

[4] Norris T , Cole TJ , Bann D , et al. Duration of obesity exposure between ages 10 and 40 years and its relationship with cardiometabolic disease risk factors: a cohort study. PLoS Med. 2020;17(12):e1003387. 33290405 10.1371/journal.pmed.1003387 PMC7723271

[5] Noncommunicable diseases: Childhood overweight and obesity World Health Organization. https://www.who.int/news-room/q-a-detail/noncommunicable-diseases-childhood-overweight-and-obesity. Accessed December 20, 2020

[6] Gluckman PD , Hanson MA . Living with the past: evolution, development, and patterns of disease. Science. 2004;305(5691):1733‐1736. 15375258 10.1126/science.1095292

[7] Rodriguez‐Caro H , Williams SA . Strategies to reduce non‐communicable diseases in the offspring: negative and positive in utero programming. J Dev Orig Health Dis. 2018;9(6):642‐652. 30111388 10.1017/S2040174418000569

[8] Nicolucci AC , Reimer RA . Prebiotics as a modulator of gut microbiota in paediatric obesity. Pediatr Obes. 2017;12(4):265‐273. 27072327 10.1111/ijpo.12140

[9] Gomes AC , Hoffmann C , Mota JF . The human gut microbiota: metabolism and perspective in obesity. Gut Microbes. 2018;9(4):308‐325. 29667480 10.1080/19490976.2018.1465157 PMC6219651

[10] Liu R , Hong J , Xu X , et al. Gut microbiome and serum metabolome alterations in obesity and after weight‐loss intervention. Nat Med. 2017;23(7):859‐868. 28628112 10.1038/nm.4358

[11] Ridaura VK , Faith JJ , Rey FE , et al. Gut microbiota from twins discordant for obesity modulate metabolism in mice. Science. 2013;341(6150):1241214. 24009397 10.1126/science.1241214 PMC3829625

[12] Kincaid HJ , Nagpal R , Yadav H . Microbiome‐immune‐metabolic axis in the epidemic of childhood obesity: evidence and opportunities. Obes Rev. 2020;21(2):e12963. 31663251 10.1111/obr.12963 PMC7771488

[13] Lee WJ , Hase K . Gut microbiota‐generated metabolites in animal health and disease. Nat Chem Biol. 2014;10(6):416‐424. 24838170 10.1038/nchembio.1535

[14] Del Fiol FS , Balcao VM , Barberato‐Fillho S , Lopes LC , Bergamaschi CC . Obesity: a new adverse effect of antibiotics? Front Pharmacol. 2018;9:1408. 30559670 10.3389/fphar.2018.01408 PMC6287021

[15] Kaliannan K , Wang B , Li XY , Bhan AK , Kang JX . Omega‐3 fatty acids prevent early‐life antibiotic exposure‐induced gut microbiota dysbiosis and later‐life obesity. Int J Obes. 2016;40(6):1039‐1042. 10.1038/ijo.2016.27 26876435

[16] Aversa Z , Atkinson EJ , Schafer MJ , et al. Association of infant antibiotic exposure with childhood health outcomes. Mayo Clin Proc. 2020;96:66‐77. 33208243 10.1016/j.mayocp.2020.07.019 PMC7796951

[17] Park YJ , Chang J , Lee G , Son JS , Park SM . Association of class number, cumulative exposure, and earlier initiation of antibiotics during the first two‐years of life with subsequent childhood obesity. Metabolism. 2020;112:154348. 32891674 10.1016/j.metabol.2020.154348

[18] Saari A , Virta LJ , Sankilampi U , Dunkel L , Saxen H . Antibiotic exposure in infancy and risk of being overweight in the first 24 months of life. Pediatrics. 2015;135(4):617‐626. 25825533 10.1542/peds.2014-3407

[19] Poulsen MN , Pollak J , Bailey‐Davis L , Hirsch AG , Glass TA , Schwartz BS . Associations of prenatal and childhood antibiotic use with child body mass index at age 3 years. Obesity (Silver Spring). 2017;25(2):438‐444. 28124504 10.1002/oby.21719 PMC5301467

[20] Mueller NT , Whyatt R , Hoepner L , et al. Prenatal exposure to antibiotics, cesarean section and risk of childhood obesity. Int J Obes. 2015;39(4):665‐670. 10.1038/ijo.2014.180 PMC4390478 25298276

[21] Leong KSW , McLay J , Derraik JGB , et al. Associations of prenatal and childhood antibiotic exposure with obesity at age 4 years. JAMA Netw Open. 2020;3(1):e1919681. 31968118 10.1001/jamanetworkopen.2019.19681 PMC6991276

[22] Jess T , Morgen CS , Harpsoe MC , et al. Antibiotic use during pregnancy and childhood overweight: a population‐based nationwide cohort study. Sci Rep. 2019;9(1):11528. 31395930 10.1038/s41598-019-48065-9 PMC6687733

[23] Wang B , Liu J , Zhang Y , et al. Prenatal exposure to antibiotics and risk of childhood obesity in a multicenter cohort study. Am J Epidemiol. 2018;187(10):2159‐2167. 29893794 10.1093/aje/kwy122

[24] Li DK , Chen H , Ferber J , Odouli R . Maternal infection and antibiotic use in pregnancy and the risk of childhood obesity in offspring: a birth cohort study. Int J Obes. 2020;44(4):771‐780. 10.1038/s41366-019-0501-2 31804609

[25] Wan S , Guo M , Zhang T , et al. Impact of exposure to antibiotics during pregnancy and infancy on childhood obesity: a systematic review and meta‐analysis. Obesity (Silver Spring). 2020;28(4):793‐802. 32129005 10.1002/oby.22747

[26] Kawamoto T , Nitta H , Murata K , et al. Rationale and study design of the Japan Environment and Children's Study (JECS). BMC Public Health. 2014;14:25. 24410977 10.1186/1471-2458-14-25 PMC3893509

[27] Michikawa T , Nitta H , Nakayama SF , et al. Baseline profile of participants in the Japan Environment and Children's study (JECS). J Epidemiol. 2018;28(2):99‐104. 29093304 10.2188/jea.JE20170018 PMC5792233

[28] Kato N , Takimoto H , Sudo N . The cubic functions for spline smoothed L, S and M values for BMI reference data of Japanese children. Clin Pediatr Endocrinol. 2011;20(2):47‐49. 23926394 10.1297/cpe.20.47 PMC3687634

[29] Revelle W . psych: Procedures for Personality and Psychological Research, Northwestern University, Evanston, Illinois, USA. Version = 1.8.12. 2018. https://CRAN.R-project.org/package=psych

[30] Sato Y , Sakurai K , Tanabe H , et al. Maternal gut microbiota is associated with newborn anthropometrics in a sex‐specific manner. J Dev Orig Health Dis. 2019;10(6):659‐666. 31106719 10.1017/S2040174419000138

[31] Uldbjerg CS , Miller JE , Burgner D , Pedersen LH , Bech BH . Antibiotic exposure during pregnancy and childhood asthma: a national birth cohort study investigating timing of exposure and mode of delivery. Arch Dis Child. 2021;106:888‐894. 33563603 10.1136/archdischild-2020-319659

[32] Delara M , McMillan DE , Nickel NC , et al. Early life exposure to antibiotics and the risk of mood and anxiety disorders in children and adolescents: a population‐based cohort study. J Psychiatr Res. 2020;137:621‐633. 33168199 10.1016/j.jpsychires.2020.11.003

[33] Konstantinidis T , Tsigalou C , Karvelas A , Stavropoulou E , Voidarou C , Bezirtzoglou E . Effects of antibiotics upon the gut microbiome: a review of the literature. Biomedicine. 2020;8(11):502. 10.3390/biomedicines8110502 PMC7696078 33207631

[34] Neuman H , Forsythe P , Uzan A , Avni O , Koren O . Antibiotics in early life: dysbiosis and the damage done. FEMS Microbiol Rev. 2018;42(4):489‐499. 29945240 10.1093/femsre/fuy018

[35] Ng KM , Aranda‐Diaz A , Tropini C , et al. Recovery of the gut microbiota after antibiotics depends on host diet, community context, and environmental reservoirs. Cell Host Microbe. 2019;26(5):650‐665.e654. 31726029 10.1016/j.chom.2019.10.011 PMC8276089

[36] Backhed F , Roswall J , Peng Y , et al. Dynamics and stabilization of the human gut microbiome during the first year of life. Cell Host Microbe. 2015;17(5):690‐703. 25974306 10.1016/j.chom.2015.04.004

[37] Kimura I , Miyamoto J , Ohue‐Kitano R , et al. Maternal gut microbiota in pregnancy influences offspring metabolic phenotype in mice. Science. 2020;367(6481):eaaw8429. 10.1126/science.aaw8429 32108090

[38] Depner M , Taft DH , Kirjavainen PV , et al. Maturation of the gut microbiome during the first year of life contributes to the protective farm effect on childhood asthma. Nat Med. 2020;26(11):1766‐1775. 33139948 10.1038/s41591-020-1095-x

[39] Baumann‐Dudenhoeffer AM , D'Souza AW , Tarr PI , Warner BB , Dantas G . Infant diet and maternal gestational weight gain predict early metabolic maturation of gut microbiomes. Nat Med. 2018;24(12):1822‐1829. 30374198 10.1038/s41591-018-0216-2 PMC6294307

[40] Ossa JC , Yanez D , Valenzuela R , et al. Intestinal inflammation in Chilean infants fed with bovine formula vs. breast milk and its association with their gut microbiota. Front Cell Infect Microbiol. 2018;8:190. 29977866 10.3389/fcimb.2018.00190 PMC6022179

[41] Moodley‐Govender E , Mulol H , Stauber J , Manary M , Coutsoudis A . Increased exclusivity of breastfeeding associated with reduced gut inflammation in infants. Breastfeed Med. 2015;10(10):488‐492. 26594906 10.1089/bfm.2015.0110

[42] Differding MK , Doyon M , Bouchard L , et al. Potential interaction between timing of infant complementary feeding and breastfeeding duration in determination of early childhood gut microbiota composition and BMI. Pediatr Obes. 2020;15(8):e12642. 32351036 10.1111/ijpo.12642 PMC7923600

[43] Avsar TS , McLeod H , Jackson L . Health outcomes of smoking during pregnancy and the postpartum period: an umbrella review. BMC Pregnancy Childbirth. 2021;21(1):254. 33771100 10.1186/s12884-021-03729-1 PMC7995767

[44] Morisaki N , Nagata C , Yasuo S , et al. Optimal protein intake during pregnancy for reducing the risk of fetal growth restriction: the Japan environment and Children's study. Br J Nutr. 2018;120(12):1432‐1440. 30394228 10.1017/S000711451800291X

[45] Nishizawa‐Jotaki S , Sakurai K , Eguchi A , Tanabe H , Watanabe M , Mori C . Association between mercury in cord serum and sex‐specific DNA methylation in cord tissues. J Dev Orig Health Dis. 2020;12:1‐8. 10.1017/S2040174420000161 32241331

[46] Campesi I , Franconi F , Montella A , Dessole S , Capobianco G . Human umbilical cord: information mine in sex‐specific medicine. Life. 2021;11(1):52. 10.3390/life11010052 PMC7828611 33451112

[47] Sheng L , Jena PK , Hu Y , Wan YY . Age‐specific microbiota in altering host inflammatory and metabolic signaling as well as metabolome based on the sex. Hepatobiliary Surg Nutr. 2021;10(1):31‐48. 33575288 10.21037/hbsn-20-671 PMC7867716

[48] Ma J , Hong Y , Zheng N , et al. Gut microbiota remodeling reverses aging‐associated inflammation and dysregulation of systemic bile acid homeostasis in mice sex‐specifically. Gut Microbes. 2020;11(5):1450‐1474. 32515683 10.1080/19490976.2020.1763770 PMC7524276

[49] Jourova L , Vavreckova M , Zemanova N , et al. Gut microbiome alters the activity of liver cytochromes P450 in mice with sex‐dependent differences. Front Pharmacol. 2020;11:01303. 33123003 10.3389/fphar.2020.01303 PMC7566554

[50] Azad MB , Vehling L , Chan D , et al. Infant feeding and weight gain: separating breast Milk from breastfeeding and formula from food. Pediatrics. 2018;142(4):e20181092. 10.1542/peds.2018-1092 30249624

[51] Cox LM , Blaser MJ . Antibiotics in early life and obesity. Nat Rev Endocrinol. 2015;11(3):182‐190. 25488483 10.1038/nrendo.2014.210 PMC4487629

