# Supplementary material for: Association between maternal antibiotic exposure during pregnancy and childhood obesity in the Japan Environment and Children's Study
Source: Pediatr Obes. 2022 Jun 24;17(11):e12956. doi: 10.1111/ijpo.12956 (PMC9787574; doi:10.1111/ijpo.12956)
Supplement: Supplementary file 1 — Table S1. Characteristics of cohort participants [file IJPO-17-e12956-s001.pdf]

Table S1 Characteristics of cohort participants

	Inclusion	Exclusion
n	56416	47646
Maternal age at delivery (years)	31.21 (4.82)	31.14 (5.36)
Maternal height (cm)	158.39 (5.24)	157.78 (5.46)
Maternal prepregnancy weight (kg)	52.66 (8.20)	53.69 (9.66)
Pre-pregnancy BMI (kg/m^2)	20.97 (2.99)	21.55 (3.64)
Birth weight (g)	3082.08 (357.26)	2867.29 (603.26)
Gestational age at delivery (weeks)	39.20 (1.05)	37.44 (4.65)

Data are presented as mean (standard deviation)

BMI, body mass index
